# Saccharine “Tabloids”

**Published:** 1888-02

**Authors:** 


### SACCHARINE "TABLOIDS."

This remarkable product of chemical science is likely to come rapidly and extensively into use, and these tabloids are of a most convenient form for ordinary domestic purposes. One tiny tabloid dropped into a cup of coffee or tea sweetens it most agreeably and innocuously, and thus the diabetic or glycosuric patient, who has been so long deprived of the pleasure of sweetening his food, can carry a little glass tube of these tabloids in his waistcoat pocket, using them as he would lumps of sugar. One tabloid is the full equivalent in sweetening power of an ordinary lump of white sugar. Saccharin is not easily soluble in water. These tabloids are, therefore, intelligently mixed with just enough alkali to make them perfectly soluble. From their extremely convenient and compact form, they will probably soon become popular for military or other purposes where bulk is a thing to be taken into consideration.—*British Medical Journal.*

